# The Value of Optic Neuritis in Medical Diagnosis

**Published:** 1894-11-10

**Authors:** William Calwell

**Affiliations:** Physician Ulster Hospital for Children, Belfast, &c.


### Medical Progress and Hospital Clinics.

*[The Editor will be glad to receive offers of co-operation and contributions from members of the profession. All letters should be addressed to* The Editor, The Lodge, Porchester Square, London, W.]

### THE VALUE OF OPTIC NEURITIS IN MEDICAL DIAGNOSIS.

By William Calwell, M.D., Physician Ulster Hospital for Children, Belfast, &c.

Ophthalmoscopic examination is difficult and unsatisfactory to those not in constant practice, and whether the general practitioner should attempt to adopt it as one of the routine methods of examination, such as auscultation is, or should relegate it to the province of the specialist, is fairly a matter of dispute. The probabilities are that eventually it will be employed by all; for the present the generality of its use will vary with the skill and opportunities of the individual. But whether a medical man is able to ascertain the presence of optic neuritis himself, or depends on the assistance of a skilled oculist, it is certainly expected of him that he understand its significance. When other signs are insufficient for forming a diagnosis he must be able to assign the proper weight to the information ascertained by himself or afforded him by the ophthalmic surgeon.

Single optic neuritis occurs with lesions of the corresponding nerve; accordingly, if we find one disc affected and the other normal, and this condition lasts, we suspect strongly some disease or injury of the nerve between the eyeball and the chiasma; it is generally due to tumour or pressure. Double optic neuritis is the commoner and more important; the inflammation in the two discs seldom advances *pari passu;* we must remember that optic neuritis is simply an inflammation of a highly organised structure; that it runs the course of inflammation, and may be acute or chronic, slight or intense, and that it leaves behind it more or less organised exudation, and a proportionate amount of destruction of nerve-fibre—the state known as optic atrophy; subsidence of the inflammation bears no relation to its development, and exacerbations may confound our prognosis.

Two great classes of disease cause double optic neuritis—intracranial and constitutional. Of the first class, intracranial tumours show it in by far the largest proportion; it occurs in four-fifths of all such tumours, but it varies with the site, occurring roughly in over 80 per cent. of basic growths, in over 40 per cent. in those of the convexity or frontal region; the involvement of brain tissue increases the percentage; confinement to the membranes alone, with pressure merely on the brain, lowers it. However, it may be absent in very large tumours, and may be present in the case of one no larger than a cherry; it may not be present at first, and may supervene at any stage; an acute neuritis, suddenly developing in the case of a sluggish tumour, points to some extension of the growth, but a chronic neuritis will not exist with a rapidly-growing one. Severe congestive double optic neuritis may be considered pathognomonic of tumour of the brain, when supported by other symptoms, and the course of the inflammation is sometimes an index to the development of the tumour.

Abscess of the brain yields a much lower percentage; probably it is somewhat more frequent than statistics show. It is more frequent with that originating from injury than with that originating in ear disease; occasionally the terminal symptoms of abscess resemble those of apoplexy; here its absence would point most strongly to the latter, its presence to the former; extra-dural abscesses are frequently accompanied by it. In meningitis reports yield great divergency of results. In ordinary tubercular meningitis of the base it is found in about half the cases, probably in more if careful and extensive examinations were made, but it comes too late to be of any real use, and no doubt both in this disease and in purulent meningitis generally, and in abscess it would be universal if the patients were to live long enough; however, it takes five or six days for optic neuritis to develop. It occurs also with thrombosis of the lateral sinus and of the cavernous sinus, with caries of the sphenoid, and with meningitis due to caries of the other bones. It may be one of the indications of a syphilitic gumma or a syphilitic meningitis; but in all these with proportions too uncertain, and with absolutely no importance as regards differential diagnosis. It points strongly to a coarse and dangerous intracranial lesion, but does not help us to answer the question, Where and what is it? It has occurred but very rarely with hydrocephalus, with aneurism, with softening from embolus, with diffuse cerebritis, with myelitis, and in insanity.

The constitutional conditions which cause optic neuritis are many and various; in typhus and puerperal fever, in pneumonia, scarlatina, alcoholism, plumbism, and sudden suppression of menses it has been found, but the instances are rare, and its presence points to some exceptional cerebral complication; it is common in chronic Bright's disease, but its existence would be rather confusing if found alone and not associated with the characteristic retinal degenerations and hæmorrhages; the presence of these latter phenomena is of importance and points to a late stage in a chronic disease when the vascular system has become affected, and a fatal issue must not be overlooked in preparing a prognosis. It has also been found in diabetes, but infrequently as compared with Bright's disease, and, as in that disease, is accompanied by retinal changes; also in anæmia, and Gowers warns us of the necessity for the administration of iron, under which treatment the neuritis subsides; if the exhibition of this drug is delayed, and, as frequently happens, owing to a suspicion of the presence of syphilis, the iodide of potassium given, the neuritis will continue till the nerve is destroyed, and atrophy will result. Independent of the direct treatment of the inflammation, suitable treatment in some of the previous diseases occasionally has a successful result as regards the ultimate fate of the nerve. Optic neuritis has subsided after the removal of a tumour, the opening and free draining of an abscess, the removal of dead bone and subsequent improvement of a localised meningitis, the administration of iodide of potassium in tertiary syphilitic cerebral manifestations; and this must not be lost sight of in the question of diagnosis.

Double optic atrophy is most commonly a sequel of double optic neuritis; its degree depends on the severity and persistence of the previous inflammation, and it requires many months for the final stage to be reached. It is highly important to remember that the yellow spot is free in optic neuritis (as it is free from connective tissue), and consequently that the acuity of vision may not be lost at the time, but that several weeks afterwards the sight may grow dimmer, till it is finally lost, and so the ophthalmoscope must be used early, and loss of vision not waited for. Double atrophy may also be primary, and is found as an early sign in locomotor ataxia, and more rarely still in one or two other nervous ailments.

We thus see that optic neuritis is not in itself a pathognomonic sign. In some diseases, of which cerebral tumour is by far the most important, it is of great help; in others it is an ambiguous or equivocal sign; in others it is misleading, and, except as a matter of pathological completeness, it might have been better had we not discovered it. Its presence points to an organic mischief within the skull, either inflammatory or pressure-causing, but it is not a localising or differentiating sign. Its absence is not conclusive.

With the ophthalmoscope we enjoy the unique advantages of seeing a nerve in a state of inflammation and the accompanying vascular changes. Probably analagous inflammation occurs elsewhere—deafness and optic atrophy after symptoms of meningitis, the slow and irregular pulse in some chronic brain lesions, and many instances of peripheral neuritis might be adduced as probable examples. Owing to the fact that the optic nerve is exceptional in its anatomy, we may be too much inclined to assign the cause of its inflammation to this peculiarity, and to suspect obscure defects in other nerves as functional, than we would find to be the truth if we could see all nerves equally as well as the optic.

